# Accessibility and Affordability of Alcohol Dependency Medical Care in Serbia

**DOI:** 10.3389/fpsyt.2014.00192

**Published:** 2015-01-12

**Authors:** Mihajlo B. Jakovljevic, Mirjana Jovanovic, Otto Michael Lesch

**Affiliations:** ^1^Department of Pharmacology and Toxicology, Faculty of Medical Sciences, University of Kragujevac, Kragujevac, Serbia; ^2^Department of Psychiatry, Faculty of Medical Sciences, University of Kragujevac, Kragujevac, Serbia; ^3^Department of Psychiatry and Psychotherapy, Medical University of Vienna, Vienna, Austria

**Keywords:** alcohol, dependency, addiction, affordability, economics, medical care, Serbia, access

## Contemporary Hot Issues on Alcohol Dependency in Serbia

Alcoholic beverages are traditional to the Balkans region since the Antiquity. Consumption of domestic honey-made spirits and wine was wide spread among the Serbs even in the early medieval history before spreading of Christianity (1). Today, it remains a deeply rooted custom while prevalence of full scale dependency and associated disorders in Serbia are slightly less frequent than European average (3.4 vs. 4.0% and 5.2 vs. 7.5%, respectively) Alcohol dependence in cited WHO sources was confirmed according to ICD-10 criteria. (2). Although hosting one of the oldest medical societies across European region, Serbia still lacks systematic morbidity and mortality registries for a variety of leading prosperity diseases including alcohol-related disorders (3). Nevertheless some national estimates on standardized death rates attributed to alcohol-related causes and alcohol consumption are available thus allowing the insight into trends of change since late 1990s.

Likewise, elsewhere in the European community, traffic accidents caused by alcohol consumption posed a substantial challenge to the road safety (4). Undisputed relationship between alcohol blood concentration and severity of acquired traffic injuries was proven in national forensic studies on cases with fatal outcome (5). Afterward, national policies have been adopted with more severe legislation framework and punitive measures compared to previous real socialism legacy (6). Unfortunately, like many other public health strategies, this one actually had quite limited effect to alcohol attributed traffic accident mortality in consecutive years (7). According to the forensic observation of a local sample of deceased with mortal injuries, craniocerebral trauma remains the most typical clinical pattern (8).

Among direct alcohol effects on mortality are classified as mental disorders with suicidal tendency (9) as well as poisoning with ethanol, methanol, and unspecified alcoholic compounds, which were attributed a total of 248 fatal outcomes in 2012. Indirect alcohol effects on mortality present far more extensive burden for the health system. Diseases such as alcohol-caused degeneration of nervous system, alcoholic cardiomyopathy, alcoholic liver disease, and alcohol-induced chronic pancreatitis caused a total of 430 deaths in 2012 according to latest official release (10). Observing the landscape of age-stratified direct alcohol-related deaths due to overdoses, one could notice heavy domination of 45–79 years age group.

According to the criminal justice procedure in Serbia, there are some 106 beds of Special Prison Hospital in Belgrade reserved for mandatory treatment of offenders who committed crimes in alcoholic condition. Such treatments are either been conducted in the penitentiary institution itself or elsewhere, in a specialized medical facility.

Quite a peculiar issue is non-fatal alcohol overdoses whose treatment lies within responsibility of the National Poison Control Center of the Military Medical Academy being the reference institution in the country for diagnostics and treatment of intoxications. This facility reported 2078 patients admitted because of acute ethylalcohol intoxications or almost 50% of all cases in 2012 (11). Broad portion of similar clinical cases, with or without suicidal intention, are being admitted to the emergency wards of other tertiary university clinics across the countries. So far illicit psychoactive substances cause several fold less overdoses than alcohol although drugs-caused mortality belongs to far younger adolescent age group (12). One promising trend among the youth is slightly decreasing prevalence of those who have ever used alcohol from 75.6% in 2008 to 64.3% level in 2012 (13). Risky attitudes of adolescents toward the alcohol use contributed substantially to the current morbidity (14). Recent pan-European trends expose binge drinking habits and co-assumption of diverse novel psychoactive substances, which contribute to the challenging nature of alcohol addiction treatment (15).

## Work Load and Economic Consequences for the National Health System

Health economics evidence is still not being deployed as mandatory tool in a routine policy making across the Balkan region (16). Gradually, the regional capacities are expanding but far from maturity to reach management staff across regional ministries of health and large university hospitals. An overall economic burden imposed by pharmaceuticals is coming to focus of expert attention due to recession-caused difficulties in health care funding (17). Globally, growing burden of mental illness including addiction disorders is no exception in South-Eastern Europe. Addictive behaviors led by alcohol abuse are frequently being tolerated because of prevailing popular opinion among the Balkan communities (14, 18). Alcohol consumption among the aged over 15 years in Serbia is around 12.6 l of pure alcohol annually, which is slightly above average for European region. Prevalence of heavy drinking among the general population was around 7.7% while among drinkers only 11.1% in 2010 (19). Heavy drinking was defined as drinking at least 60 g or more of pure alcohol on at least one occasion in the past 30 days based on WHO recommendations.

Lesch Alcoholism Typology (LAT) as an interactive software containing treatment guidelines has been introduced in seven major regional addiction centers throughout Serbia. It distinguishes among four major subtypes of alcohol-dependent patients. The first one is “allergy model” (craving caused by alcohol); the second one “conflict resolution and anxiety model” (craving caused by stress); the third “depressive model” (craving caused by mood); and the fourth is known as the “conditioning model” (craving caused by compulsion). An important feature of alcohol-dependent patients in Serbia is significantly higher anxiety levels and clear clinical domination of LAT type III. Major underlying reasons for this phenomenon might be the post-traumatic stress syndrome among almost one million refugees and internally displaced persons suffering from consequences of civil wars in former Yugoslavia back in 1990s. Another key issue is increased poverty levels due to global recession. The increased anxiety presence among the addictive patient population should be regarded as an important local feature of clinical presentation uncommon to many high-income EU communities. Such morbidity structure is inevitably reflected to demand for medical services including psychotherapy and pharmaceuticals (20). An essential determinant of local clinical setting is involuntary nature of patient treatment and detoxification. In majority of cases, patients are brought to specialized clinic on civil order violation charges by police or by the emergency medicine vehicle after being found in a severely intoxicated state. This is quite different compared to the decently high degree of patient awareness for the necessity of treatment in many developed societies (21).

Regardless of quite strong legacy of alcohol abuse and dependence, national capacities for necessary medical care remain insufficient. Long-term-based residential alcoholism rehabilitation covers <10% (22). Although it is believed that up to 50% of patients receive either in- or outpatient detoxification in case of need, this service is actually one of core weaknesses of the national network of medical facilities, primarily specialist psychiatry clinics, which are in charge of their care (23). The local evidence points out to the huge proportion of almost 50% legal enforcement expenses related to the prosecution of citizens committing variety of criminal offenses due to harmful alcohol use. Based on field assessments conducted on a local cohort of patients, an estimate on urgent need of up to €9,784,011 investment into expanding existing network of detoxification facilities has been published (24). Efforts have been made toward development of draft document on national strategy to combat alcohol addiction in Serbia by Committee for Psychoactive Controlled Substances of the Ministry of Health, which is likely to undergo official voting procedure in the upcoming period. Growing consciousness among policy makers and the citizens is supported by variety of initiatives by governmental agencies, district institutes of public health, regional addiction centers, and NGOs. Legal framework for the associated activities was established by adopting the Law on Psychoactive Controlled Substances (25) as well as several other laws and directives coordinated with the EU legal framework (26).

## Challenge of Medical Care Affordability to the Ordinary Citizen

Serbia’s health system has passed a long way from former municipally owned health care funded through mixed-Bismarck model of former Yugoslavia. It should not be forgotten that the largest market of Western Balkans, due to historical circumstances, actually entered socioeconomic transition with one decade long delay compared to most of Eastern Europe (27). Its achievements through the past 15 years of successive and bold health reforms have been substantial in some areas. The key ongoing difficulty ultimately affecting provision and delivery of care to alcohol-dependent patients is heavy burden of out-of pocket expenses for the ordinary citizens (28). This is particularly worsened by delayed marketing approval of some leading anti-craving medicines and their poor and ineffective market access mostly due to reimbursement limitations in place. There are up to 37% of LAT type I and II patients likely to benefit substantially from acamprosate (29) and up to 63% of LAT type III and IV patients in Serbia to whom naltrexone is indicated (30). It is sad reality that even <5% of Serbian patients for whom acamprosate or naltrexone was indicated were actually prescribed and dispensed these drugs in a recent national trial (20). The major obstacle remains too narrow reimbursement criteria and poor affordability of medicines. This situation is emphasized with recession-worsened employment rates in domestic market, increased taxes, and large portion of households sinking below poverty level (31).

**Figure 1:**
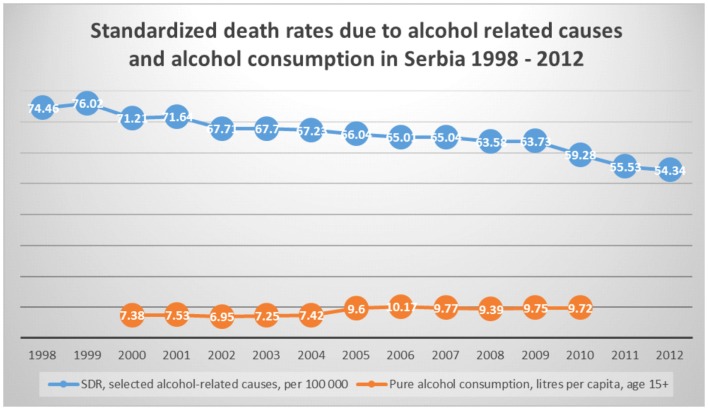
**Standardized death rates and alcohol consumption in Serbia 1998–2012**. *Source of data – European Health for All Database (HFA-DB) (31).

One of the promising trends observed in time series of cross sectional data on the region reported by the national authorities to European Health for All Database (HFA-DB) (32) is steadily decreasing standardized death rate due to alcohol-attributable causes (Figure 1). This is opposed to the slightly upward trend in the overall annual alcohol consumption across the country. There is a variety of ground changes in the health system to explain this surprising phenomenon. Among others, it is improved responsiveness of psychiatric care in treatment of mood disorders such as alcohol-induced depression associated with suicidal tendency in its clinical presentation. Others might be better quality and accessibility of neurological and hepatology-related care whose cost-efficiency in some cases was actually approved by local evidence (33).

Another intriguing local developments were related to the official implementation and staff training in LAT software and its clinical guidelines in seven major regional addiction centers in Serbia since 2011 (34). After 2 years of its application in clinical practice by the local addictologists and psychiatrists, it turned out to generate cost savings for the system in a matched-pairs case–control study design (35). These and similar advances are promising for the future of alcohol addiction prevention, medical care, and rehabilitation in Serbia.

At contemporary momentum, it appears that accessibility and affordability of alcohol dependency medical care remain far from satisfactory levels in Serbia. Efficient new financing instruments will be required to fund existing network of specialty addiction centers as well as to provide reimbursement of anti-craving medicines. There is a long way ahead. Current support from ground professional associations such as ESBRA (12) and EU institutional commitment (36) seem to provide optimistic prospects in this area even for the small Balkan communities awaiting EU accession.

## Conflict of Interest Statement

No conflict of interest with regards to the manuscript disclosed by Mihajlo B. Jakovljevic, Mirjana Jovanovic, and Otto Michael Lesch.
